# Fluctuating Hyperthyroidism and Hypothyroidism in Graves’ Disease: The Swinging Between Two Clinical Entities

**DOI:** 10.7759/cureus.27715

**Published:** 2022-08-05

**Authors:** Aakriti Shrestha, Namrata Adhikari, Saujan Devkota, Tutul Chowdhury, Zewge Shiferaw-Deribe, Nicole Gousy, Samaj Adhikari

**Affiliations:** 1 Internal Medicine, Interfaith Medical Center, Brooklyn, USA; 2 Internal Medicine/Endocrinology, Interfaith Medical Center, Brooklyn, USA; 3 Medicine, American University of Antigua, New York City, USA

**Keywords:** thyroid peroxidase antibodies, hashimoto's hypothyroidism, hypothyroid, hyperthyroid, graves's disease

## Abstract

Autoimmune thyroid disorders are frequently encountered in clinical practice and consist of a spectrum ranging from Graves’ hyperthyroidism to Hashimoto's hypothyroidism. Generally, patients with autoimmune thyroid disorders will lean towards one end of the spectrum or the other, with fluctuations between hyper- and hypothyroidism rarely seen. This is especially the case when persistent hyperthyroidism occurs after a prolonged period of hypothyroidism. Here, we present a case of a young female patient initially presenting with Graves’ disease with a previous history of hypothyroidism.

## Introduction

Graves’ disease and Hashimoto's thyroiditis are two autoimmune diseases of the thyroid that present on opposite ends of a spectrum. Graves’ disease is usually associated with hyperthyroidism, while Hashimoto's thyroiditis is usually associated with hypothyroidism. The fluctuation of hyperthyroidism and hypothyroidism is a rarer phenomenon with fewer published case reports [1-3]. Antibodies responsible for these two states are thyroid-stimulating antibodies and thyroid-blocking antibodies, respectively. The balance between these antibodies determines thyroid functionality. We present a case of a young female with an initial hypothyroid state followed by a hyperthyroid state in Graves’ disease.

## Case presentation

**Figure 1:**
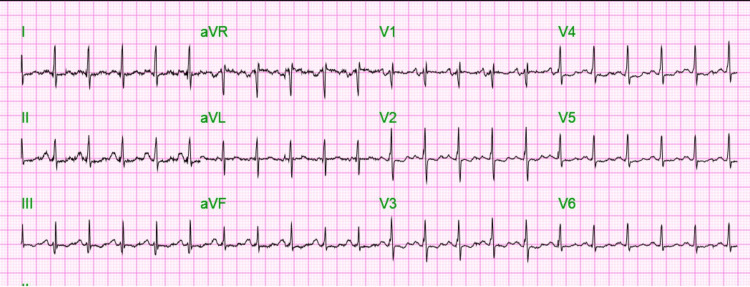
Admission EKG showing sinus tachycardia

A 45-year-old African American female presented with a past medical history of chronic obstructive pulmonary disease (COPD) requiring 3-5 L of home oxygen, heart failure with preserved ejection fraction, pulmonary embolism (2016 and 2018), pulmonary hypertension, obstructive sleep apnea requiring nightly continuous positive airway pressure (CPAP), and hyperthyroidism. She presented to the emergency department in June 2022 with right-sided chest pain and shortness of breath for a week. Triage vitals in the emergency room were as follows: blood pressure was 134/89 mmHg, pulse was 125/min, temperature was 98.7°F, and SpO_2_ was 95% on nasal cannula 3 L. She was found to be tachycardic and in mild respiratory distress (Figure 1).

She was admitted for acute exacerbation of COPD and managed with IV steroids, a bronchodilator, antibiotics, and supplemental oxygen. Given her history of hyperthyroidism, a thyroid panel was obtained and the patient was resumed on a home dose of methimazole 10 mg daily. During her endocrinology evaluation, the patient endorsed that she was initially diagnosed with hypothyroidism three years ago and was treated with levothyroxine. A thyroid panel in August 2020 showed a thyroid-stimulating hormone (TSH) of 58 and a free T4 level of 0.12. She was treated with levothyroxine 50 mcg daily during this time. However, in June 2021 during previous hospital admission for bronchitis, she displayed features of hyperthyroidism including heat intolerance, palpitations, increased defecation to two bowel movements per day, and a weight loss of 10 lbs. A thyroid panel done during that admission showed a TSH of < 0.015, and a free T4 of 2.62, consistent with hyperthyroidism, therefore levothyroxine was discontinued. However, after discontinuation of the levothyroxine, she remained in a hyperthyroid state and was started on methimazole 10 mg at another facility in April 2022. When she presented to our facility during the current admission, she complained of loss of appetite, unquantified weight loss, and bulging of her eyes. On physical examination, she was noted to have mild exophthalmos, tachycardia, and a normal thyroid examination.

Investigations, diagnosis, and treatment

**Figure 2:**
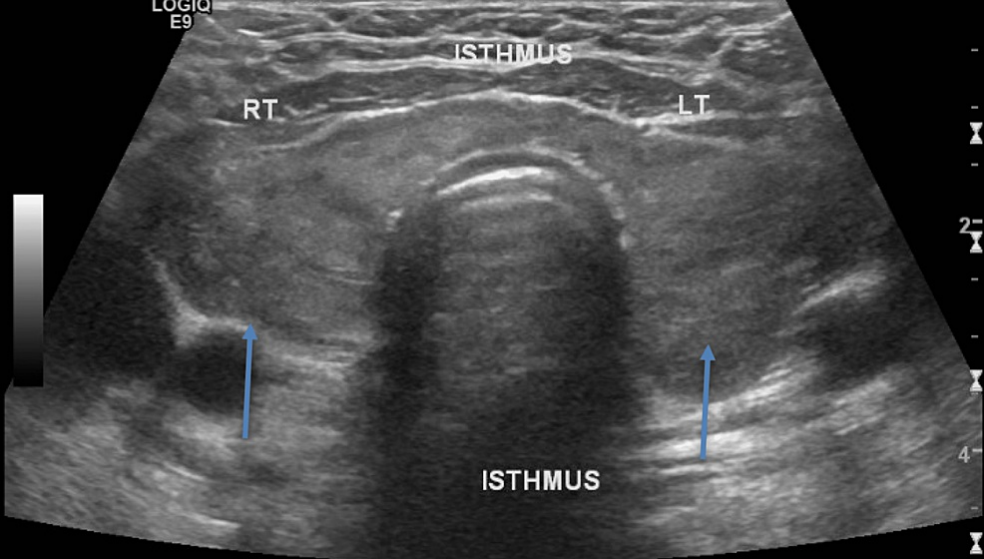
Ultrasound of the thyroid and parathyroid showing right and left thyroid lobes along with isthmus Of note is the heterogeneous thyroid with a nodule on the right as well as left sides (arrows).

A thyroid and parathyroid ultrasound was done that measured the right thyroid lobe as 4.8 x 1.8 x 1.9 cm. There was also a 1.9 x 1.0 x 1.6 cm heterogeneous slightly hypoechoic nodule in the lower pole of the right thyroid. The left thyroid lobe measured 4.3 x 2.1 x 3.5 cm, with a 2.2 x 1.4 x 2.1 cm heterogeneous slightly hypoechoic nodule in the mid pole of the left thyroid lobe also noted. The thyroid isthmus thickness was 0.68 cm, within the normal range (Figure 2).

**Figure 3:**
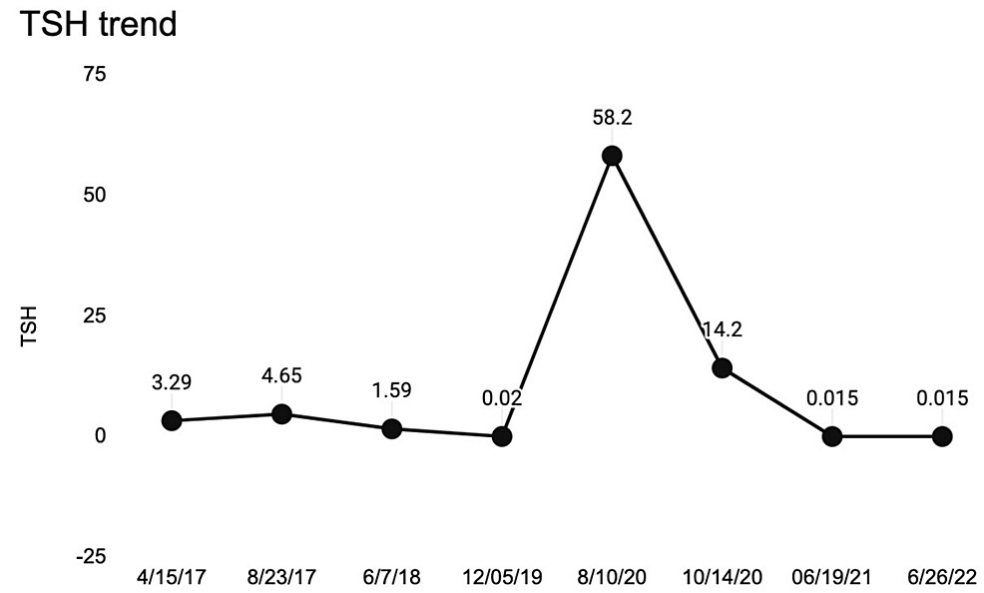
TSH trend pointing to gradual transformation from hypothyroid to hyperthyroid state TSH: thyroid-stimulating hormone

A thyroid panel during this admission was consistent with hyperthyroidism with TSH <0.015, a free T4 was 3.32, and a T3 level of 293 (Table 1). Additional testings for thyroid antibodies were all positive (Figure 3). Unfortunately, there were no available values for thyroid peroxidase antibodies from her hypothyroid state.

**Table 1:** Thyroid panel showing hyperthyroidism with positive antibodies TSH: thyroid-stimulating hormone

Component	Value	Ref range and units
TSH	< 0.015	0.465-4.680 uIU/mL
Free T4	3.32	0.78-2.19 ng/dL
T3	293	71-180 ng/dL
TSH receptor binding inhibitory immunoglobulin (TSB Ab)	4.63	0.00-1.75 IU/L
Thyroid peroxidase antibodies (TPO)	300	0-34 IU/mL
Thyroid-stimulating immunoglobulin (TSI or TS Ab)	5.86	0.00-0.55 IU/L

Methimazole was subsequently increased to 20 mg daily with the addition of propranolol 20 mg daily for tachycardia. A CT angiogram of the chest was done and pulmonary embolism was ruled out. The patient improved symptomatically and her tachycardia resolved.

The patient was offered definitive treatment including radioactive Iodine and thyroidectomy, however, she refused to pursue this treatment method. She was discharged home on methimazole and propranolol to be followed up in the endocrinology clinic as an outpatient.

## Discussion

Autoimmune thyroid disorder consists of Hashimoto’s thyroiditis and Graves’ disease. We have seen many previously reported cases with Graves' hyperthyroidism switching to hypothyroidism. Previously, it was believed that patients with Grave’s disease had only thyroid-stimulating hormone (TSH) antibodies. However, it has been recognized that thyroid-stimulating antibody (TS Ab) and thyroid-stimulating blocking antibody (TSB Ab) can be present in the same patient [1]. It has been noted that the TS Ab, which can be potent at lower concentrations, can induce hyperthyroidism initially. However, it has been observed that disease progression through the continuous expression of TSB Ab, and thyroid peroxidase antibody (TPO) can induce lymphocytic infiltration of the thyroid gland, therefore inducing follicle destruction. Thus leading to eventual hypothyroidism in the clinical presentation [1].

It is a very rare phenomenon to see conversion from hypothyroidism to Grave’s hyperthyroidism [4]. This could be attributed to the increased levels of TPO that are relatively associated with incurring massive lymphocytic infiltration, fibrosis and thyroid follicle damage, leading to a progressive, irreversible hypothyroid state [4]. When irreversible cellular damage and fibrosis occur in the thyroid, it is then difficult to undergo extensive follicular regeneration to the point of inducing a hyperthyroid state. We present the rarest switch from hypothyroidism to Grave’s hyperthyroidism. 

It is believed that these conversions are associated with three factors: TS Ab in blood, TSB Ab in blood, and the response of the thyroid gland to TS Ab [5]. Graves’ hyperthyroidism is caused by TS Ab and hypothyroidism is caused by TSB Ab [6]. The switch between the TS Ab and TSB Ab is more commonly seen in women [7]. It was also seen in some studies that initially hypothyroid patients with TSB Ab treated with levothyroxine switched to TS Ab and hyperthyroidism [4,8]. These conversions are also seen with anti-thyroid drug use and normal physiological changes in pregnancy. As the concentrations of these antibodies switch, the clinical presentation varies.

Our patient was also on levothyroxine treatment during her hypothyroid phase of presentation. It has been noted in some studies that levothyroxine treatment might cause a decrease in thyroid autoantibodies but in unusual cases, thyroid autoantibody levels can increase [7]. We believe this mechanism could be one of the causes of swinging presentations in our patient. While the test for antibodies was not available in our case report when the patient was in the hypothyroid phase, another possible explanation would be that the initial hypothyroidism could be from transient thyroiditis rather than the chronic destructive Hashimoto’s thyroiditis. It can also be attributed to the thyroid-stimulating hormone receptor (TSHR) antibody blockage before the TS Ab concentration reached the critical level needed to induce hyperthyroidism. These changes can be seen because of differences in different individual patients related to affinities, relative concentration, and potencies.

These alterations in the antibodies pool and differences in the clinical presentation of the patients make it difficult to stabilize the thyroid hormone replacement in the long term. Replacement or blocking therapy is useful temporarily. It would require regular thyroid function tests and follow-up for the stability of thyroid hormone levels. For long-term stability, definitive therapy including both thyroidectomy and radioactive iodine is useful. However, these interventions need patients in the hyperthyroid phase. Our patient also presented in the hyperthyroid phase, but the offered definitive treatment was refused by the patient. She was discharged on anti-thyroid drugs and is planned to follow-up in the endocrinology clinic after discharge.

## Conclusions

Swinging between hyperthyroidism and hypothyroidism due to spontaneous oscillation in thyroid function is an infrequent clinical scenario. The flipping from hypothyroidism to hyperthyroidism invites the challenge of managing a patient with Graves’ disease in a definitive manner. Since most patients with Graves’ disease receive thyroidectomy or radioactive iodine, this alternate functioning phenomenon is uncommonly encountered in our daily practice. Herein, we presented a patient who initially presented with hypothyroidism and later showed contrasting findings of hyperthyroidism highlighting the rare phenomenon of switching between TSH receptor-stimulating antibodies and TSH receptor-blocking antibodies.
